# Effects of effortful swallowing on cardiac autonomic control in individuals with neurogenic dysphagia: a prospective observational analytical study

**DOI:** 10.1038/s41598-020-67903-9

**Published:** 2020-07-02

**Authors:** Livia M. S. Gomes, Roberta G. da Silva, Cristiane R. Pedroni, David M. Garner, Rodrigo D. Raimundo, Vitor E. Valenti

**Affiliations:** 1 Department of Speech, Hearing and Language Pathology, UNESP, Marilia, Brazil; 2 Department of Physical Therapy and Occupational Therapy, UNESP, Marilia, Brazil; 3 Cardiorespiratory Research Group, Department of Biological and Medical Sciences, Faculty of Health and Life Sciences, Oxford Brookes University, Headington Campus, Oxford, OX3 0BP UK; 4 Laboratório de Delineamento de Estudos e Escrita Científica, Centro Universitário Saúde ABC, Avenida Príncipe de Gales, 667, Bairro Príncipe de Gales, Santo André, São Paulo CEP: 09060-590 Brazil

**Keywords:** Cardiology, Neurology

## Abstract

Considering that neurogenic oropharyngeal dysphagia is a prevalent condition with or without cardiac disease we should contemplate issues surrounding cardiovascular difficulties during rehabilitation. This study aims to evaluate the effects of effortful swallowing maneuver (ESM) on heart rate variability (HRV) in subjects with neurogenic oropharyngeal dysphagia. We studied 22 individuals [8 Stroke and 14 Parkinson Disease (PD) subjects aged between 41 and 75 years old] with neurogenic oropharyngeal dysphagia regardless of gender. HRV was assessed under two circumstances: spontaneous swallowing versus ESM. Surface electromyography of the suprahyoid muscles was undertaken to measure the swallowing muscle excitation, which then confirmed higher muscle activity during ESM. We attained no changes in HRV between the two swallowing events [HR: spontaneous swallowing 78.68 ± 13.91 bpm vs. ESM 102.57 ± 107.81 bpm, p = 0.201; RMSSD (root-mean square of differences between adjacent normal RR intervals in a time interval): spontaneous swallowing 16.99 ± 15.65 ms vs. ESM 44.74 ± 138.85 ms, p = 0.312; HF (high frequency): spontaneous swallowing 119.35 ± 273 ms^2^ vs. ESM 99.83 ± 194.58 ms^2^, p = 0.301; SD1 (standard deviation of the instantaneous variability of the beat-to-beat heart rate): spontaneous swallowing 12.02 ± 1.07 ms vs. ESM 31.66 ± 98.25 ms, p = 0.301]. The effortful swallowing maneuver did not cause clinically significant changes in autonomic control of HR in this group of subjects with oropharyngeal dysphagia.

## Introduction

The occurrence of neurogenic oropharyngeal dysphagia is frequently observed in lesions that affect the central or peripheral nervous system, including strokes, neurodegenerative diseases, traumatic brain injury and cerebral palsy^1,2^. Consequently, recurrent pneumonia, malnutrition and excessive hospital expenses impact the quality of life of patients with and without the condition^3^.

In an attempt to treat these problems, different techniques of swallowing training protocol are started for rehabilitation. Swallowing protocols aim to improve swallowing functions and the individuals’ quality of life^4^. Hence, Logemann and Kahrilas^5^ (1990) first described the effortful swallowing maneuver (ESM). This is a maneuver necessary to intensify the forces during swallowing, contributing to the tongue base movement and anterior movement of the pharyngeal wall, which results in increased oral propulsion and reduced oral and pharyngeal residue^5^. One of the physiological changes during effortful swallowing are chiefly because of the suprahyoid muscles’ contraction, which involves the anterior belly of the digastric, mylohyoid and geniohyoid muscles^6^.

In the scientific research literature, the impact of ESM on autonomic cardiac function is seldom discussed. Previous studies have reported details of swallowing-induced tachycardia^7,8,9^. The physiological mechanism proposed to explain this is founded on the stimulation of the left atrium because of the distended esophagus, inducing an adrenergic reflex from the esophagus and vasovagal reflex^10^.

Of late, a study assessed cardiac autonomic regulation via heart rate (HR) variability (HRV) in healthy subjects throughout spontaneous swallowing^11^. The researchers revealed sudden distinct losses in the inter beat intervals (RR intervals) during the spontaneous swallowing stages. Frequency domain HRV analysis was related under three circumstances: spontaneous swallowing *vs.* without spontaneous swallowing *vs*. spontaneous swallowing eliminated. It was revealed that HRV was influenced by spontaneous swallowing, increasing vagal regulation of HR.

We investigated the acute effects of ESM on HRV in young healthy women in 2016^12^. HRV was evaluated for five minutes during spontaneous swallowing vs. effortful swallowing. The effortful swallowing maneuver reduced the high frequency (HF) band and increased the low frequency/high frequency (LF/HF) ratio, suggesting that these maneuvers negatively influence cardiac autonomic regulation in young females.

Given that reduced HRV is related to higher risks of cardiovascular complications^13^, the aforesaid findings were concerning. This is since the effortful swallowing maneuver is a system included in many rehabilitation protocols required in individuals with cardiovascular disorders associated with oropharyngeal dysphagia^1^. So, we endeavored to investigate the effects of effortful swallowing on cardiac autonomic control in individuals with neurogenic oropharyngeal dysphagia.

## Results

Table 1 presents all comorbidities for the patients included.

**Table 1:** All comorbidities for the patients included.

Diseases	N
Parkinson disease	6
Stroke	4
Stroke and Parkinson and hypertension	1
Parkinson and prostate cancer	1
Parkinson and diastolic dysfunction	1
Parkinson and bladder obstruction	1

**Figure 1:**
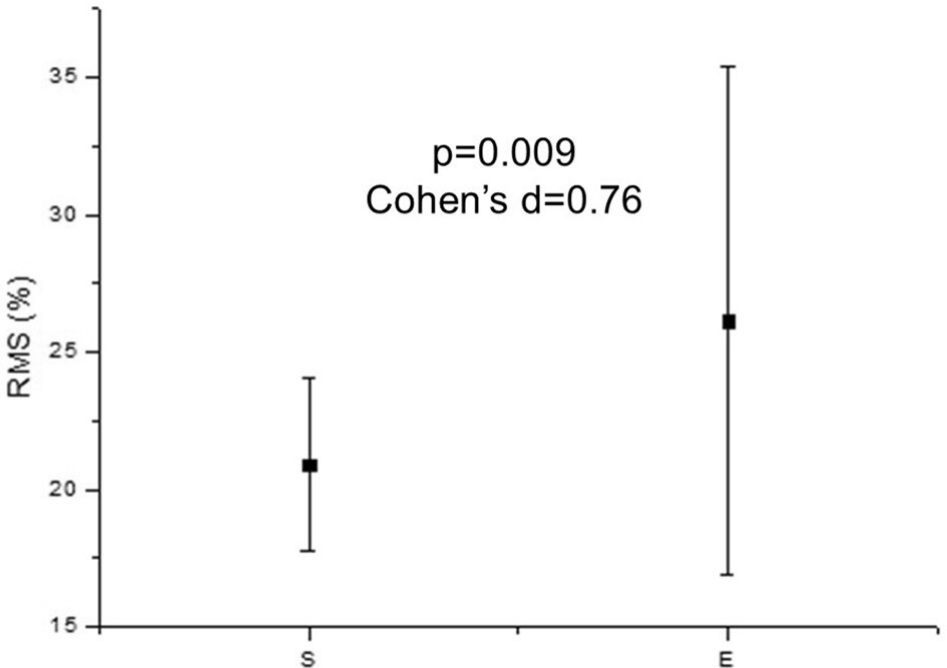
Mean values and standard deviation of RMS of all subjects. *RMS* root mean square.

Figure 1 illustrates increased muscle activity during effortful swallowing compared to spontaneous swallowing.

**Figure 2:**
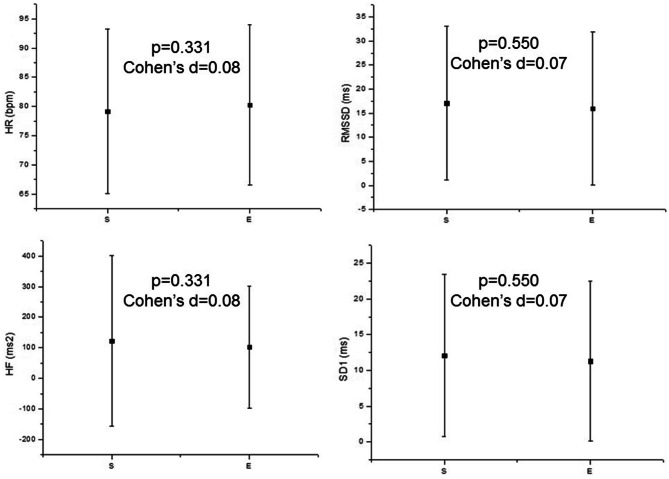
Mean values and respective standard deviation of mean HR, RMSSD, HF and SD1 indexes during spontaneous and effortful swallowing. *HR* heart rate, *RMSSD* square root of the square mean of the differences between adjacent normal RR intervals, *SD1* standard deviation of instantaneous beat-to-beat variability, *HF* high frequency, *S* spontaneous swallowing, *E* effortful swallowing.

Figure 2 illustrates HRV during spontaneous and effortful swallowing. We observed no changes in mean HR, RMSSD, HF and SD1 indexes.

In Table 2, we observed moderate and negative correlation between delta EMG and delta HRV.

**Table 2:** Correlation between delta EMG and delta HRV indices.

HRV indices	r	p
RMSSD	− 0.73	0.0001
HF	− 0.43	0.04
SD1	− 0.73	0.0001

*RMSSD* square root of the square mean of the differences between adjacent normal RR intervals, *SD1* standard deviation of instantaneous beat-to-beat variability, *HF* high frequency

Table 3 presents data concerning mean and standard deviation of age, height, mass, body mass index (BMI), systolic (SBP) and diastolic blood pressure (DBP).

**Table 3:** Mean and standard deviation of age, height, mass, BMI, SBP and DBP.

Variables	Mean (SD)
Age (years)	66.24 (9.53)
Height (m)	1.65 (0.1)
Mass (kg)	68.3 (12.7)
BMI (kg/m^2^)	25.24 (4.2)
SBP (mmHg)	11.84 (1.18)
DBP (mmHg)	70.2 (2.24)

*m* meters, *g* grams, *BMI* body mass index, *mmHg* millimeters of mercury, *SBP* systolic blood pressure, *DBP* diastolic blood pressure, *SD* standard deviation.

## Discussion

Up till now, we have shown that ESM reduced vagal control of HR^12^ in healthy females, inducing cardiac overload. Although the cited study investigated only women to discard the influence of sexual hormones, this outcome permitted us to hypothesize that effortful swallowing would be problematic for individuals with neurogenic oropharyngeal dysphagia. Hence, we considered the acute effects of ESM on HR autonomic regulation in subjects with neurogenic oropharyngeal dysphagia. As a key conclusion, based on a small sample and individuals with stroke and Parkinson disease we suggested that HRV was not clinically different between spontaneous and effortful swallowing. We also revealed an association between swallowing muscle excitation changes during ESM and HRV changes during this procedure.

Not all subjects with oropharyngeal dysphagia were capable of achieving a change between effortful and spontaneous swallowing regarding swallowing strength. Therefore, it may have changed the relationship with HRV, because this group may lack the required strength.

We calculated HRV through well-recognized indices, represented by RMSSD, HF and SD1. RMSSD corresponds to time domain analysis, HF is a frequency domain index and SD1 represents the Poincaré plot^13^. All indices provide evidence about the influence of the parasympathetic nervous system on heart rhythm^21^.

Therefore, the interaction between cardiac autonomic regulation and swallowing is founded on the parasympathetic nervous system activity^22^. The parasympathetic fibers that exit the vagus nerve are accountable for cardiovascular autonomic regulation^23,24^. The myelinated portion of the vagus nerve provides efferent control of the heart through regulation of the sinus node^25^ and this has an impact on HRV during respiratory sinus arrhythmia; when HR increases during inspiration owing to vagal withdrawal and decreases during expiration because of vagal recovery^26^.

The trigeminal and vagus nerves perform a central role regarding the interaction between swallowing and cardiac autonomic control. Both nerves are linked to the innervation of the larynx and heart muscles^27^. A study conducted by Kecskes et al.^27^ employed a confocal laser scanning microscope and investigated brainstem areas involved with the parasympathetic activity and the swallowing phase. These authors advise that motor neurons of nucleus ambiguus and trigeminal afferent terminals are linked for the coordination of muscles related to swallowing phase. Similarly, trigeminal nerve and the nucleus ambiguus are involved in the heart’s rhythm^28,29^. So, it is expected that swallowing influences the HRV.

Conversely, it is rare to find in the scientific research literature studies related to the impact of swallowing on the heartbeat’s oscillations, which is a benchmark of heart activity^13^. Numata et al. (2013)^30^ examined nine healthy men, which completed 13 intentional swallows, once every 30 s amongst expiration and inspiration. During the experimental protocol, the authors recorded RR intervals. It was detected that swallowing-induced tachycardia recovered within one breath cycle. The above-mentioned outcome permits us to consider if HRV is influenced by the five minutes of effortful swallowing maneuvers.

To resolve this, in an earlier study^12^, we assessed 34 healthy females that performed effortful swallowing whilst recording RR intervals. It was revealed that HRV decreased during effortful swallowing compared to spontaneous swallowing. It was recommended that respiratory rate was an important variable related to HR autonomic changes induced by swallowing^31^. Gomes et al.^12^ intended to remove potential sources of bias to provide a better physiological interpretation. Instead, our investigation provides results with improved clinical application, since the subjects examined are from the ambulatory routine.

Next, Yildiz and Doma^11^ (2018) studied the acute influence of spontaneous saliva swallowing on HRV. The authors evaluated 13 young female and 17 young male subjects. It was detailed that impulsive saliva swallowing influences HRV.

Together, the cited references indicate that the effortful swallowing rehabilitation technique should be carefully performed in subjects with dysphagia associated to cardiac injuries. Yet, our up-to-date results reveal that HRV during effortful swallowing was not clinically different in subjects with neurogenic dysphagia. This data demonstrates the safety of this rehabilitation technique for subjects with neurogenic dysphagia.

There are some mechanisms that could help us to explain the slight effect of ESM on HRV changes in individuals with neurogenic dysphagia examined here, in this study. One option is that swallowing strength is greater in healthy individuals and so it would induce more powerful autonomic responses. We^12^ (2016) and Yildiz and Doma^11^ (2018) assessed healthy young individuals, whilst here we considered older individuals (> 41 years old). It is well recognized that tongue strength decreases with aging^32^, yet, the influence of tongue strength and swallowing in healthy older adults is unclear.

A further possibility is the participation of nutritional status in swallowing strength. Our study population presented higher BMI compared to the investigation previously conducted in our laboratory^12^. Nutritional status evaluated through BMI and its relationship with swallowing has been previously investigated^33^. The cited study was performed on individuals between 20 and 40 years old according to BMI (obese: 30 ≤ BMI < 35, overweight: 25 ≤ BMI < 30, normal range: 18.5 ≤ BMI < 25 and underweight: 17 ≤ BMI < 18.5). The results did not support an exact style of eating, while it was revealed that injury in masticatory function in those obese individuals may be related to swallowing strength.

We emphasize some points in our study to describe the potential sources of bias regarding HRV influences. We did not evaluate the LF band of spectral analysis and the LF/HF ratio. This is because the LF/HF ratio has been repeatedly confirmed to be theoretically flawed and empirically unsupported. Though there are many criticisms of the quantity, the most concerning aspect is that LF does not index sympathetic activity, and so there is a lack of rationale and evidence that its strength in relation to the HF component would index relative strength of vagal and sympathetic signaling^36–38^.

To standardize the circadian influences on HRV, we accomplished all experiments in the morning (between 8:00 and 11:59). It is important to mention this as Melatonin has a powerful effect on HR autonomic regulation^34,35^. Temperature and humidity were controlled, as they influence autonomic function^39^. Standard deviation was greater than 9.3 ms as a result of the higher variation of HRV between individuals. Thus, according to strategies for HRV experiment planning, data analysis, and data reporting from Laborde et al.^40^, within-subject design has more advantages compared to the between-subject comparison. This is since within-subject design contributes to the elimination of individual differences in respiratory rates and reduces the impact of external variables, including medication, alcohol, smoking, and so forth. Medication could be a variable that had an influence on HRV and contributed to standard deviation > 9.3 ms.

Many subjects in this study presented with Parkinson disease. Parkinson's disease is known to present dysautonomia^41^ and, as a result, decreased HRV^42^. One may theorize that individuals with Parkinson would present different responses to autonomic stimulation compared to stroke subjects. However, after a careful search on the Medline/PubMed database, we were unable to find any suggestion that provide differences between Parkinson and stroke individuals regarding HRV changes induced by autonomic tests.

We cannot reject the influence of medication on HRV during ESM, since Parkinson subjects were undergoing Levodopa treatment.

Small sample size is an important limitation to be addressed, which makes the study underpowered. As a result of a rigorous exclusion criteria, we evaluated 22 individuals with neurogenic dysphagia. Consistent with the paired Student t-test assuming alpha = 0.05, the power of the study is 38%.

If we included patients who were unable to perform the manoeuver, the power of the study to detect significant impact of ESM on HRV would increase. Yet, rigor of potential sources of bias would be weakened, increasing the chance of equivocal physiological interpretations.

Under this scenario, we recommend further experimentations to confirm our results.

Our findings are significant to the clinical protocols that undertake dysphagia rehabilitation interventions. We suggest that the effortful swallowing rehabilitation technique does not induce substantial cardiovascular risk in individuals with neurogenic dysphagia. Still, the small sample size highlights the need for additional studies to support this hypothesis. Thus far, we are unable to clarify if the procedure is entirely safe for individuals with dysphagia accompanying a preceding myocardial infarct. Therefore, the effortful swallowing maneuver did not cause deviations in the autonomic control of HR in individuals with neurogenic oropharyngeal dysphagia.

## Conclusion

Based on a small sample comprised of individuals with stroke and Parkinson disease, our results advised that the effortful swallowing maneuver did not cause clinical changes in the autonomic control of HR in individuals with neurogenic oropharyngeal dysphagia. Further experimental research studies are required to confirm our results.

## Methods

### STROBE guidelines

This study conforms to the STROBE (STrengthening the Reporting of OBservational studies in Epidemiology) guidelines. Our investigation provides details of the study design, surroundings, participants, variables, data sources, measurement, description of potential sources of bias, quantitative variables description, and statistical methods.

### Population study and eligibility criteria

**Figure 3:**
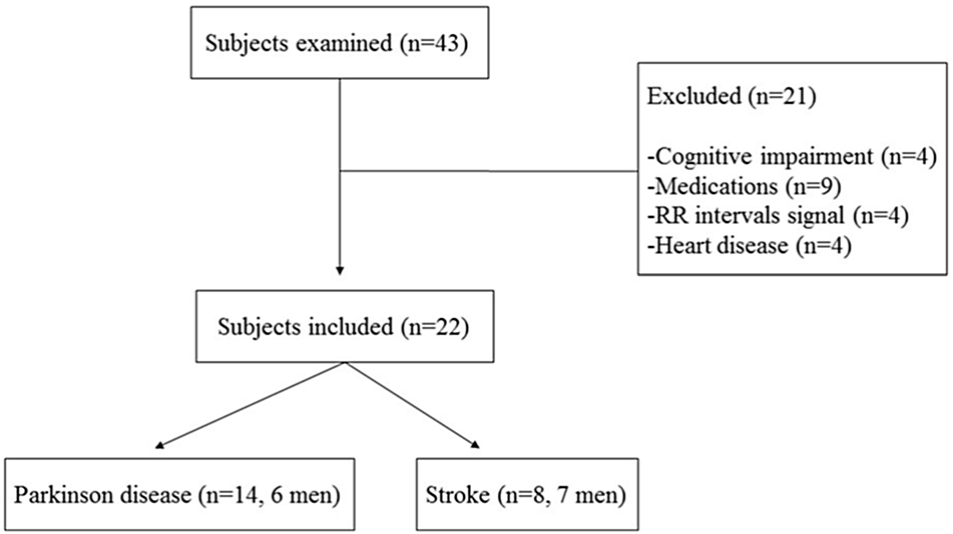
Flow chart describing individuals excluded.

We principally evaluated 43 individuals with ischemic Stroke and Parkinson Disease of both genders and aged between 41 and 90 years (mean: 67 years). We excluded individuals under pharmacotherapies, apart from individuals with Parkinson Disease that were undergoing treatment with Levodopa. We likewise excluded individuals unable to perform ESM, individuals with cardiac arrhythmias, respiratory, endocrine diseases and other impairments that circumvented them performing all the requirements of the study. Similarly, individuals with cognitive impairments incapacitating the comprehension of guidelines required for the tasks and more than 5% of artifacts during RR intervals recording. Hence, we omitted 22 individuals (Fig. 3).

The validation of the presence of oropharyngeal dysphagia was accomplished via the clinical evaluation of swallowing, achieved by thorough anamnesis and clinical evaluation, and objective evaluation through fiberoptic endoscopy assessment of swallowing. This was commenced to consider etiological, general clinical aspects, in addition to the subjects’ performance whilst feeding.

### Study design and setting

This is a prospective, observational and analytical study performed at the Laboratory of Dysphagia at Sao Paulo State University.

### Bias

We accomplished all experimental protocols in the same room with temperature between 20 and 25 °C and humidity between 40 and 60% to anticipate potential sources of bias. HRV was assessed at the same time, in the morning (between 8:00 and 11:59) to standardize circadian influences. The subjects were directed to avoid drinking caffeine and ingesting other autonomic stimulants for 24 h prior to experiments and to sustain an empty bladder during the HRV analysis.

With the intention of lessening the variables unpredictability, improve reproducibility and physiological clarification, individuals were recognized according to age, mass (kg), height (m), systolic (mmHg) and diastolic arterial pressure (mmHg) and body mass index (BMI).

### Initial assessment

The individuals established a diagnostic protocol to document oropharyngeal dysphagia from a specialist Dysphagia Research Rehabilitation Center.

Confirmation of the presence or absence of dysphagia was completed through a clinical swallowing examination, detailed anamnesis, with the objective of investigating etiological, and wide-ranging clinical features, besides the individual’s current performance whilst feeding. A clinical swallowing assessment was achieved using indirect and direct swallowing evaluations by clinical observations.

After the clinical evaluation, individuals were submitted to an objective evaluation via a fiberoptic endoscopy swallowing inspection. We considered oropharyngeal dysphagia as the presence of at least one of the subsequent outcomes: presence of posterior oral spillage, pharyngeal residues, laryngeal penetration and/or laryngotracheal aspiration.

Before the beginning of the experimental procedure, the subjects were documented by collecting the following data: age, gender, mass, height, BMI, systolic (SBP) and diastolic blood pressure (DBP).

The anthropometric dimensions were reached according to the recommendations described by Lohman et al.^14^. The BMI was calculated using the following mathematical formula: mass (kg)/height (m)^2^. The authentication of SBP and DBP occurred indirectly, using a stethoscope and aneroid sphygmomanometer on the left arm. To evade errors whilst determining the subjects blood pressures, the same researcher measured these cardiovascular parameters throughout.

Table 2 displays the mean and standard deviation of the individuals, characterizing the sample according to age, height, mass, body mass index (BMI), SBP and DBP.

### Experimental protocols

The ESM training protocol was started according to knowledge from the Dysphagia Research Rehabilitation Center team in the Department of Speech, Language and Hearing Sciences, based on the instructions proposed by Huckabee and Steele^15^ (2006). The specific protocol concerned three effortful swallows, each one in 1 min and 30 s during 5 min. The subjects were coached to complete the maneuver through tongue force on the palate, which intends to intensify the tongues force and pressure during swallowing, improve coordination and decrease the posterior movement of the base of the tongue^15^.

The experimental procedures were split into two randomized stages, via card allotments on the same day:

(a) Spontaneous swallowing: The subjects remained at rest for five minutes. They undertook three spontaneous swallows of saliva under command every 1 min and 30 s, and were instructed to swallow the saliva at other periods, if needed, as is typically the case.

(b) Effortful swallowing: Following the initial five minutes, subjects performed the training effortful swallowing technique, as before, similarly as was the case to all subjects, for an additional five minutes. The subjects completed three effortful swallowing, every 1 min 30 s minutes as directed.

HRV was equated by spontaneous swallowing *vs.* effortful swallowing protocol^12^.

### Variables, data sources and outcome measures

#### HRV analysis

We recorded RR intervals through the RS800CX HR monitor, which had a sampling rate of 1 kHz. The RR intervals were transferred to the Polar Precision Performance program (v.3.0, Polar Electro, Finland). The Polar HR monitor distinguishes all heart beats in the left ventricular cardiac muscle and records the data, transferring the signal to the computer through a wireless infrared methodology. The Polar ProTrainer software allows the visualization and the removal of the RR interval file in “txt” format.

Specifics of the HRV analysis have been supplied previously^16,17^ and were in accordance with the directives from the Task Force^13^.

To study the parasympathetic regulation of HR we inspected the high frequency band of spectral analysis (HF: 0.15 Hz to 0.4 Hz) in absolute units in the frequency domain, RMSSD (root-mean square of differences between adjacent normal RR intervals) in the time domain and the SD1 Poincaré plot (standard deviation of the instantaneous variability of the beat-to-beat heart rate). We applied the Kubios^®^ HRV v. 2.0 software to compute these indices^18^.

#### Electromyographic evaluation

With the purpose of authenticating the swallowing strength, the individuals were assessed through surface electromyography to measure the amplitude ​​of the electrical activity in suprahyoid muscles.

A six-channel active filter with analog bandpass filters and a cutoff frequency of 20–1000 Hz was required and scanned with 16 bits of resolution and simultaneous sampling of the signals using a Lyxelectrograph (Lynx Tecnologia LTDA, Sao Paulo, SP, Brazil). The equipment was connected to a battery with a capacity of 10 A hour, potential difference of 12 V, and to a computer without a connection to the electrical mains system to avoid interference.

Aqdados (Lynx^®^) software, version 7.02 for Windows, was required for data acquisition and storage of the scanned signals. The electrodes were passive surface silver chloride, disposable self-adhesive with 20 mm distance between the poles (Miotec^®^) linked via cables with an alligator-type clip, coupled in a preamplifier of Lynx Tecnologia LTDA, with input impedance of 10 G, and the common mode rejection rate (CMRR) of 130 dB with a gain of 20.

A circular stainless-steel electrode, 3 cm diameter, smeared with conductive ice, was applied as a reference to diminish signal acquisition noise.

The datasets were studied in the time domain by Root Mean Square (RMS) values, which were then normalized by their maximum values of the first isometric contraction.

The visualization and digital processing of the electromyographic signal was completed via the AqDAnalysys of Lynx Tecnologia Ltda program. All activities regarding the recording and assessment of the electromyographic signal conformed to the Standards for Reporting EMG Data^19^. Electrical signals were normalized using the maximum value of the first effort contraction.

### Study size

We conducted post-hoc sample size calculations via online software at https://www.lee.dante.br. We considered the RMSSD index, the significant difference in magnitude expected was 12.8 ms, with a standard deviation of 9.3 ms; per alpha risk of 5% and the power of the test 80%. The sample size provided a minimum of 21 volunteers per group.

### Statistical analysis

We performed a Shapiro–Wilk normality test to assess the distributions. To compare HRV during spontaneous swallowing vs. effortful swallowing, we executed the paired Student t-test for parametric distribution and the Wilcoxon test for non-parametric distributions. Statistical significance was considered for p < 0.05 (or < 5%).

To estimate the magnitude of changes between spontaneous swallowing vs. effortful swallowing we considered the effect size through Cohen’s *d*. Large effect size was considered for values greater than 0.9, medium for values between 0.9 and 0.5 and small for values less than 0.5^20^.

So as to evaluate the association between a change in EMG (ESM minus baseline) and a change in HRV parameters (delta = at ESM minus at baseline), we completed a non-parametric Spearman correlation test between delta RMS vs. delta RMSSD, delta RMS vs. delta HF and delta RMS vs. delta SD1. We considered strong correlations for r greater than 0.75 and moderate correlations for r between 0.75 and 0.5.

### Ethical approval and informed consent

All subjects were informed about procedures and objectives of the study and, after agreeing, signed a confidential informed consent letter. The study was accepted by the Research Ethics Committee in Research of Paulista State University, (Number 1.503.509). All procedures were performed in accordance with the relevant guidelines and regulations, statement explicitly stated that the methods were in accordance with the 466/2012 resolution of the National Health Council of 12/12/2012.

## Supplementary information

Supplementary file 1
